# Left ventricular global longitudinal strain in predicting CRT response: one more J-shaped curve in medicine

**DOI:** 10.1007/s00380-021-01770-w

**Published:** 2021-02-06

**Authors:** Michal Orszulak, Artur Filipecki, Wojciech Wrobel, Adrianna Berger-Kucza, Witold Orszulak, Dagmara Urbanczyk-Swic, Wojciech Kwasniewski, Katarzyna Mizia-Stec

**Affiliations:** First Department of Cardiology, School of Medicine in Katowice, Medical University of Silesia, ul Ziolowa 45/47, 40-635 Katowice, Poland

**Keywords:** CRT, Left Ventricular Global  Longitudinal Strain, LVEF, Heart failure

## Abstract

The aim of the study was: (1) to verify the hypothesis that left ventricular global longitudinal strain (LVGLS) may be of additive prognostic value in prediction CRT response and (2) to obtain such a LVGLS value that in the best optimal way enables to characterize potential CRT responders. Forty-nine HF patients (age 66.5 ± 10 years, LVEF 24.9 ± 6.4%, LBBB 71.4%, 57.1% ischemic aetiology of HF) underwent CRT implantation. Transthoracic echocardiography was performed prior to and 15 ± 7 months after CRT implantation. Speckle-tracking echocardiography was performed to assess longitudinal left ventricular function as LVGLS. The response to CRT was defined as a ≥ 15% reduction in the left ventricular end-systolic volume (∆LVESV). Thirty-six (73.5%) patients responded to CRT. There was no linear correlation between baseline LVGLS and ∆LVESV (*r* = 0.09; *p* = 0.56). The patients were divided according to the percentile of baseline LVGLS: above 80th percentile; between 80 and 40th percentile; below 40th percentile. Two peripheral groups (above 80th and below 40th percentile) formed “peripheral LVGLS” and the middle group was called “mid-range LVGLS”. The absolute LVGLS cutoff values were − 6.07% (40th percentile) and − 8.67% (80th percentile). For the group of 20 (40.8%) “mid-range LVGLS” patients mean ΔLVESV was 33.3 ± 16.9% while for “peripheral LVGLS” ΔLVESV was 16.2 ± 18.8% (*p* < 0.001). Among non-ischemic HF etiology, all “mid-range LVGLS” patients (100%) responded positively to CRT (in “peripheral LVGLS”—55% responders; *p* = 0.015). Baseline LVGLS may have a potential prognostic value in prediction CRT response with relationship of inverted J-shaped pattern. “Mid-range LVGLS” values should help to select CRT responders, especially in non-ischemic HF etiology patients.

## Introduction

Cardiac resynchronization therapy (CRT) is approved form of treatment for patients with heart failure with reduced ejection fraction (HFrEF) and prolonged QRS duration [1, 2]. Identification of ‘responders’ and ‘nonresponders’ before CRT implantation is still the essence of the matter and up to 40% of patients do not benefit after CRT implantation [3]. The incidence of non-responders remains the same for many years despite the numbers of trials and great effort that have been dedicated to improve the identification of responders. Dyssynchrony has been thought to be the missing link. Unfortunately, to date, all the approaches have turned out to be suboptimal in this regard [4].

Therefore, we hypothesized that factors other than dyssynchrony may contribute to CRT response. Strain constitutes a sensitive method for quantifying global left ventricular function [5] and can provide prognostic information beyond routine LVEF [6]. Echocardiographic strain by speckle-tracking precisely characterizes left ventricle performance [7], scar burden [8], and dyssynchrony [9]. Left ventricle global longitudinal strain (LVGLS) is associated with the outcome in general population [10] as well as in heart failure patients [11]. Previous studies have demonstrated that baseline left ventricle function stays in relation with final reverse remodeling. In one report from a post hoc analysis of the randomized control MADIT-CRT trial, LV functional improvement as assessed by ΔLVGLS was powerfully linked to an improved outcome on top of ΔLVEF or CRT response assessed by ΔLVESV [12]. Advantages of strain assessment include obtainable in most of cases and higher reproducibility than LVEF [13, 14].

The main goals were to verify if LVGLS may be of additive prognostic value in CRT response prediction and to obtain such a LVGLS value that in the best possible way enables to characterize potential CRT responders.

## Materials and methods

### Population

The prospective study included 49 patients (84% male, 66.5 ± 10 years, 34.7%/63.3% in New York Heart Association class II/III) with symptomatic heart failure who met the criteria for CRT implantation in class I/IIa according to the 2013 ESC guidelines [15]. Exclusion criteria were acute coronary syndrome for three months, inadequate CRT delivery after follow-up (BiV pacing rate < 90%) or the poor image quality of the echocardiography.

Primarily, consecutive 71 patients were enrolled into the study. Six patients were excluded due to poor echocardiographic windows, four patients were excluded because of suboptimal pace delivery (BiV < 90%) during follow-up, four patients were lost during follow-up, four patients declined to participate in the study, two patients were excluded due to dysfunction of CRT (dislocation of LV lead), and 2 patients died during follow-up. The remaining 49 patients formed a study group.

Twenty (40.8%) patients had already had a cardiac implantable electronic device (CIED) and received an upgrade to a resynchronization system, while the others (29 patients, 59.2%) received a CRT de novo. Almost all the devices had a defibrillator capability (CRT-D) and the LV was preferably placed in either the lateral or the posterolateral vein. Patients were receiving optimal pharmacological therapy.

All the patients were informed and signed a written consent. The study protocol was approved by the local bioethical committee.

### Follow-up

Patients were evaluated in one-year and long-term follow-up.

In the 1-year follow-up, a clinical status and transthoracic echocardiography were assessed; the response to CRT (“responder”) was defined in the short-term follow-up as a ≥ 15% LVESV reduction.

In the long-term follow-up, the prognostic value of the results was verified. Outcome was defined as an all-cause mortality. Vital status or information about death date were extracted from medical reports or received from patients’ relatives. Status was ascertained in December 2020.

### Echocardiography

Standard transthoracic 2D echocardiography was performed by an experienced echocardiographer using a cardiovascular ultrasound system (Vivid 7, GE Medical Systems) before and 15 months after CRT implantation. LV end-systolic volume (LVESV), end-diastolic volume (LVEDV), and LVEF were measured using the biplane Simpson method. LVGLS measurements were performed to assess the global left ventricular function.

All the measurements were performed in three representative cardiac cycles and then averaged. Echocardiographic recordings were analyzed blinded to clinical data. Image analysis was performed offline using a customized software package (EchoPac). The response to CRT was defined as a ≥ 15% LVESV reduction.

### LVGLS measurements

By two-dimensional speckle-tracking echocardiography, longitudinal strain values were obtained from three apical views with frame rates > 40 Hz. Longitudinal strain values were computed after determining aortic valve closure (AVC). AVC was determined from pulsed-wave Doppler of the LV outflow tract and was superimposed on the strain waveforms. Automatic tracking of the endocardial contour on an end-systolic frame was carefully verified. The regions of interest (ROI) were manually adjusted to be in accordance with the actual thickness of myocardium and to ensure optimal tracking. The software automatically divided myocardium into 18 segments. Strain values and the longitudinal time-strain curves for all 18 segments were computed. LVGLS was calculated as an average from the 18 segmental strain values from the three apical views.

### Statistical analysis

Statistical analysis was performed using Statistica 10 Software. Distribution was verified using the Shapiro–Wilk test. Continuous variables are expressed as the mean ± standard deviation and were compared using *U*-Mann test or Wilcoxon test or Kruskal–Wallis test when appropriate. Categorical variables (reported as numbers with percentages) were tested using *χ*^2^ statistics. Spearman rank coefficient tests were used to determine the relationships between the variables. For survival analysis, Kaplan–Meier curve with testing using log-rank statistics was performed. Reproducibility was assessed for strain measurements. Twenty studies were reanalyzed by the same and other observer to assess intra- and inter-observer variabilities. *p* value < 0.05 was considered to be statistically significant.

## Results

### Baseline characteristics

The study included a total of 49 patients (84% male, 66.5 ± 10 years, NYHA II/III/IV: 34.7%/63.3%/2%; 57.1% ischemic aetiology of HF), who underwent CRT implantation. The mean QRS duration was 173.1 ± 19.1 ms. Thirty-five (71.4%) patients had a native LBBB according to conventional ECG criteria, seven (14.3%) patients had a dominant right ventricular pacing rhythm, and seven patients had non-LBBB. The baseline characteristics are presented in Table 1. Thirty-six (73.5%) patients were CRT responders (≥ 15% reduction in LVESV) and 13 (26.5%) non-responders. There were no significant differences in the baseline demographics, clinical or echocardiographic parameters between responders and non-responders.

**Table 1:** Baseline characteristics of general population, responders, and non-responders

	Study population (*n* = 49)	Responders (*n* = 36)	Non-responders (*n* = 13)
Age (years)	67 ± 10	68 ± 10	63 ± 10
Male sex, *n* (%)	41 (84)	30 (83.3)	11 (84.6)
NYHA functional class	2.8 ± 0.5	2.8 ± 0.6	2.7 ± 0.4
Baseline NYHA class III, *n* (%)	31 (63.3)	21 (58.3)	10 (76.9)
Ischemic etiology of HF, *n* (%)	28 (57.1)	20 (55.6)	8 (61.5)
QRS (ms)	173 ± 19	173 ± 21	174 ± 16
LBBB, *n* (%)	35 (71.4)	27 (75)	8 (61.5)
AF at implantation, *n* (%)	7 (14.3)	6 (16.7)	1 (7.7)
CIED before CRT (= upgrade to CRT), *n* (%)*	20 (40.8)	11 (30.6)	9 (69.2)
LVESV (ml)	218 ± 109	217 ± 107	223 ± 119
LVEF (%)	25 ± 6	24 ± 6	27 ± 7
LVGLS	− 6.94 ± 2.16	− 6.75 ± 1.65	− 7.48 ± 3.19

*p < 0.05 responders vs. non-responders

The inter- and intra-observer variabilities for the LVGLS were 7 and 5%, respectively.

### One-year follow-up

The mean follow-up was 14.9 ± 7 months. Overall, NYHA class decreased from 2.8 ± 0.5 to 1.9 ± 0.7 (*p* < 0.001). During the follow-up, almost all the echocardiographic parameters improved. The LVEDV and LVESV volumes were reduced (from 282 ± 129 ml to 238 ± 120 ml and from 218 ± 109 ml to 166 ± 98 ml, respectively; *p* < 0.001 for both). The LVEF increased from 25.9 ± 6% to 32 ± 79%; *p* < 0.001. For the entire population, CRT led to significant increase of LVGLS: − 6.94 ± 2.16 to − 7.95 ± 2.68%; *p* = 0.039. Improvements in the echocardiographic parameters (LVESV, LVEDV, LVEF, and strain values) concerned mainly responders while in non-responders no significant changes were observed after CRT implantation.

### LVGLS and CRT response

There was no linear correlation between baseline LVGLS and ∆LVESV (*r* = 0.09; *p* = 0.56). We have found significant association between ∆LVESV and improvement in LV performance expressed by ∆LVGLS (*r* = 0.32; *p* = 0.028).

### LVGLS in prediction of CRT response

**Fig. 1:**
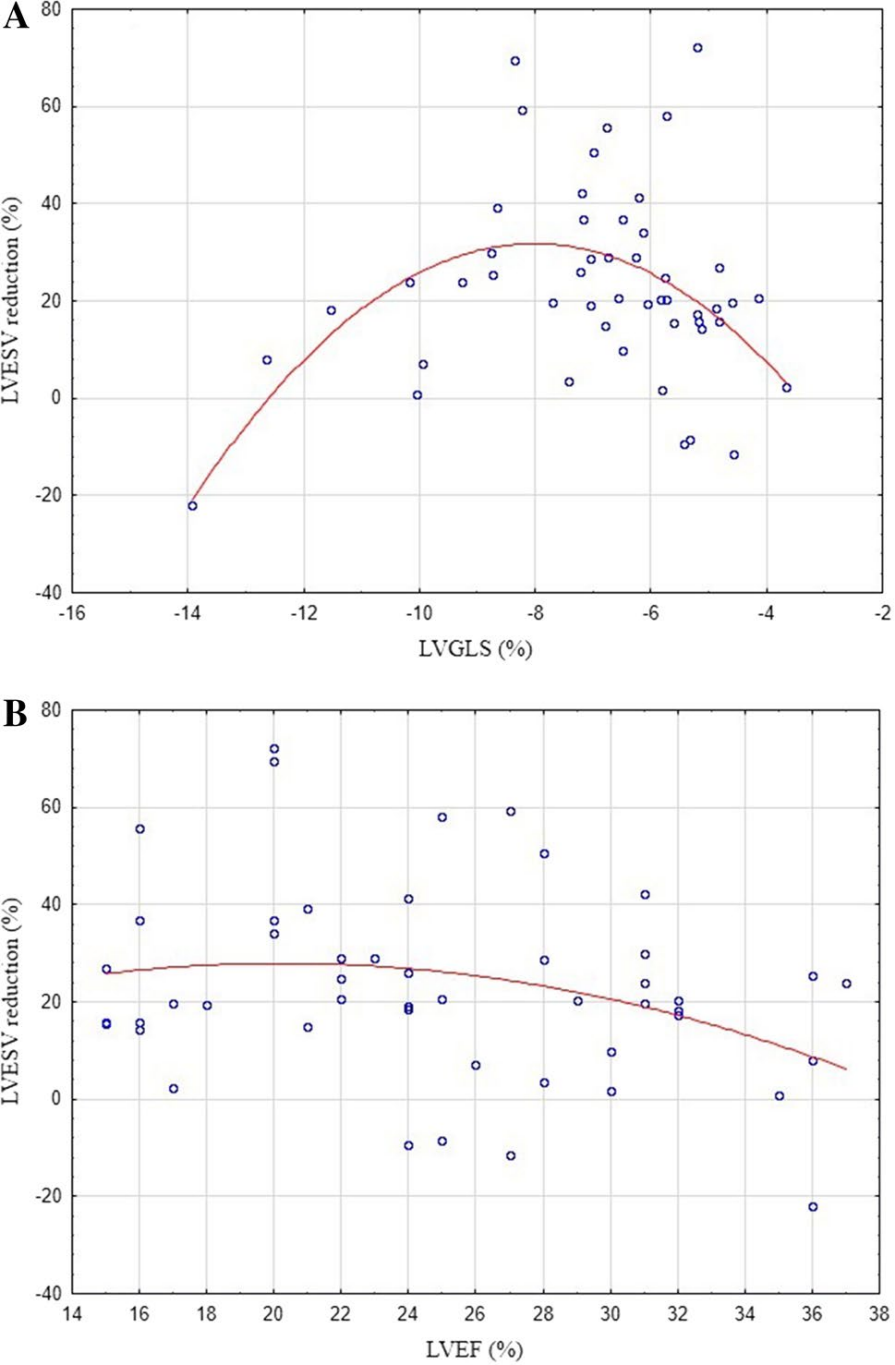
Relationship between ∆LVESV and baseline LV function assessed by LVGLS (**a**) and LVEF (**b**)

**Fig. 2:**
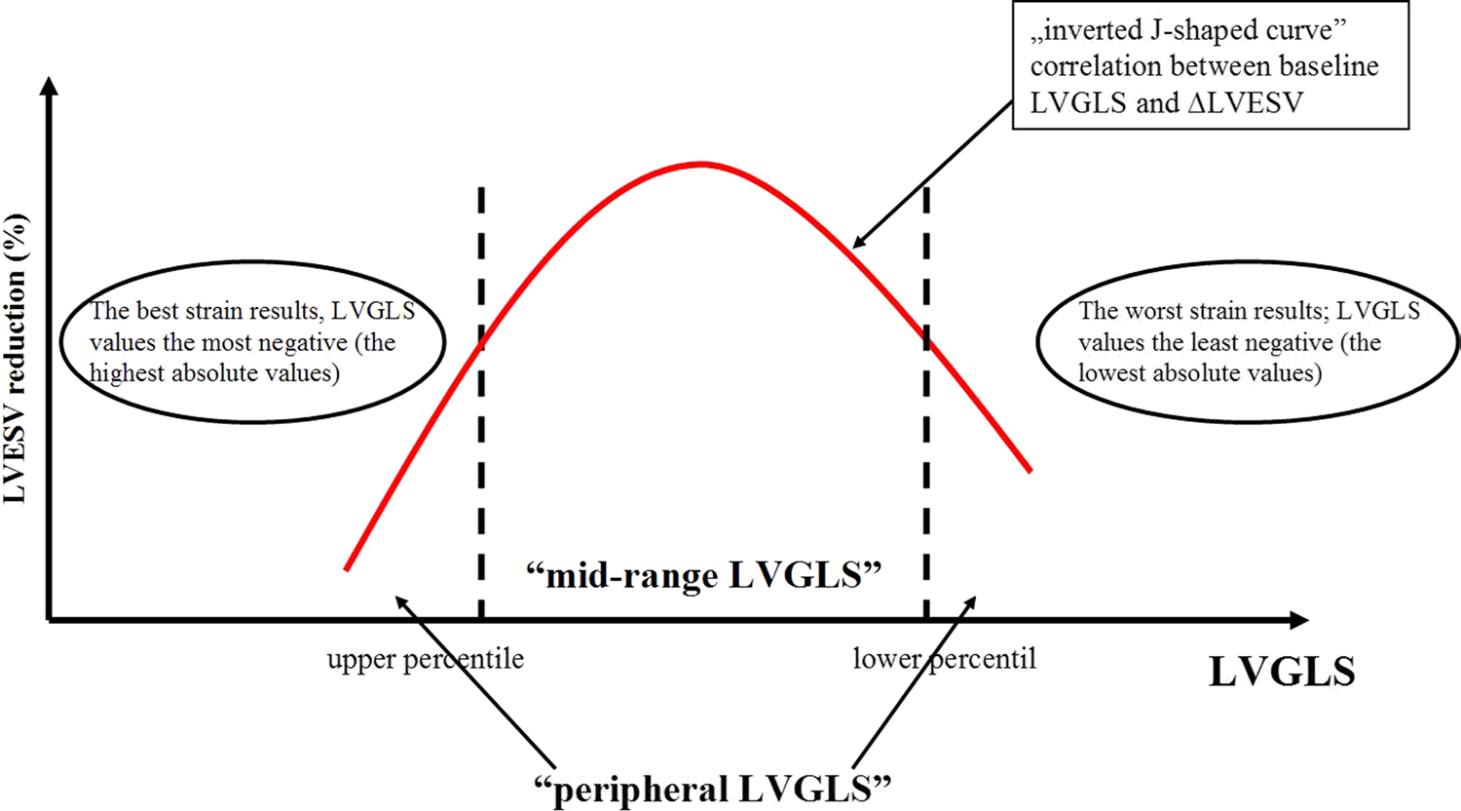
Inverted J-shaped curve relationship between baseline LVGLS and ΔLVESV. Schematic presentation of how study population was divided on “peripheral LVGLS” and “mid-range LVGLS” according to the percentile of baseline LVGLS

Correlation between baseline LVGLS and ∆LVESV was not linear (*r* = 0.09; *p* = 0.56) but it has turned out to be an inverted J-shaped curve (Fig. 1a). We sought that patients with LVGLS values corresponding to the peak of the curve might have greater chance to CRT response than the patients with the most extreme LVGLS values. Therefore, the study group was categorized into two groups: patients with middle LVGLS values (so-called “mid-range LVGLS”) and extreme LVGLS values (so-called “peripheral LVGLS”) (Fig. 2).

**Fig. 3:**
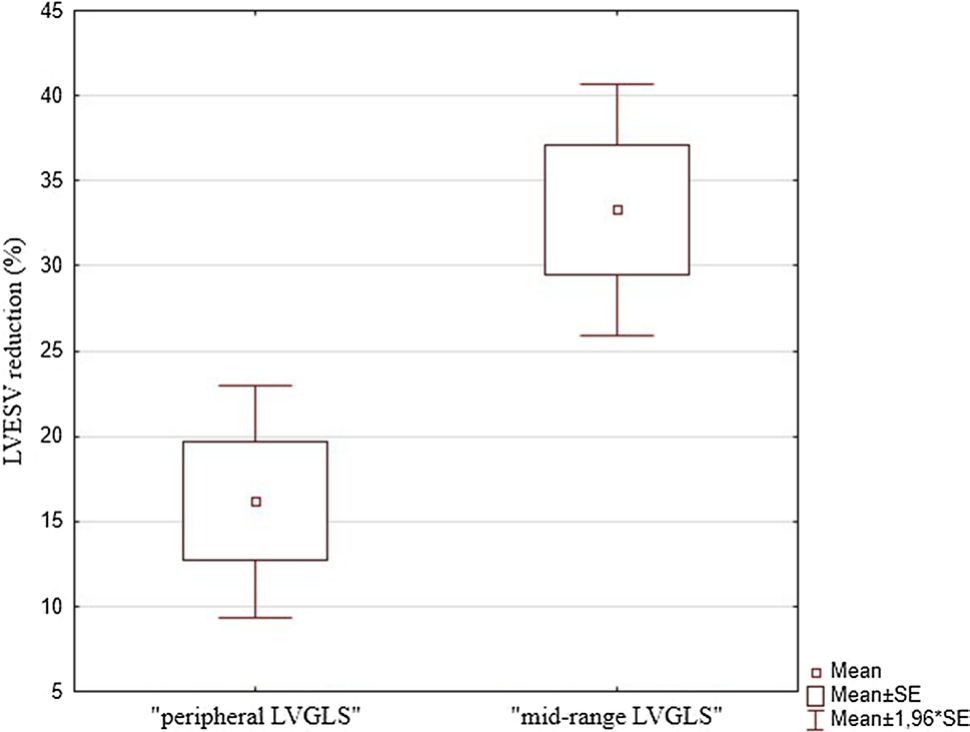
Mean ΔLVESV in “peripheral LVGLS” and “mid-range LVGLS”

Groups were formed according to the percentile of baseline LVGLS values. Few LVGLS percentile cutoff threshold were verified (Table 2) to select the most potent CRT recipient (based on ∆LVESV). The best cutoff percentile LVGLS values were obtained for 40th percentile (absolute LVGLS − 6.07%) and 80th percentile (absolute LVGLS − 8.67%). Detailed characteristics of the groups are presented in Table 3. Finally, patients with LVGLS values above 80th and below 40th percentile formed “peripheral LVGLS” while patients with LVGLS values between 80 and 40th percentile formed “mid-range LVGLS”. Characteristics of both groups are shown in Table 4. For “mid-range LVGLS” mean ΔLVESV was 33.3 ± 16.9% and for “peripheral LVGLS” 16.2 ± 18.8%. This difference was statistically significant (*p* = 0.00056) (Fig. 3). Baseline LVEF did not differ between groups: “mid-range LVGLS” 24 ± 4.5% vs. “peripheral LVGLS” 25.6 ± 7.4%; *p* = 0.4. After follow-up period, increase of LVEF (ΔLVEF) was greater in “mid-range LVGLS” than in “peripheral LVGLS” group (absolute ΔLVEF 11.2 ± 6.2% vs. 4.1 ± 8.2%; *p* = 0.0008; percentage ΔLVEF 151.3% vs. 124.4%; *p* = 0.0048). Therefore, final LVEF was higher in mid-range group than in peripheral group (35.1% vs. 29.7%; *p* = 0.0025).

**Table 2:** ∆LVESV in each groups of patients divided according to different LVGLS percentile cutoff threshold

LVGLS percentile cutoff and corresponding absolute LVGLS	Group A (the worst LVGLS values)	Group B (“mid-range LVGLS”)	Group C (the best LVGLS values)	*p* value
P_60_ = − 7% P_20_ = − 5.2%	19.3 ± 20.4% (*n* = 11)	24.4 ± 19.1% (*n* = 19)	24.2 ± 20.8% (*n* = 19)	A vs. B→ *p* = 0.27 A vs. C→ *p* = 0.22 B vs. C→ *p* = 1 B vs. (A + C)→ *p* = 0.59
P_75_ = − 5.43% P_25_ = − 7.7%	14.9 ± 21.4% (*n* = 13)	27.4 ± 14.9% (*n* = 24)	23.6 ± 24.9% (*n* = 12)	A vs. B→ *p* = 0.014 A vs. C→ *p* = 0.25 B vs. C→ *p* = 0.56 B vs. (A + C)→ *p* = 0.058
P_66_ = − 7.17% P_33_ = − 5.76%	18.4 ± 21.4% (*n* = 17)	28 ± 14.4% (*n* = 16)	23.4 ± 22.5% (*n* = 16)	A vs. B→ *p* = 0.053 A vs. C→ *p* = 0.29 B vs. C→ *p* = 0.47 B vs. (A + C)→ *p* = 0.12
P_80_ = − 8.67% P_40_ = − 6.07%	17.7 ± 19.9% (*n* = 20)	**33.3 ± 16.9%** **(n = 20)**	12.8 ± 16.3% (*n* = 9)	A vs. B→ *p* = 0.029 A vs. C→ *p* = 1 B vs. C→ *p* = 0.06 **B vs. (A + C)**→** p = 0.00056**

**Table 3:** Comparison of the groups according to 80th and 40th percentile of baseline LVGLS value

	A The worst LVGLS (below 40th percentile) *n* = 20	B “mid-range LVGLS” (between 80 and 40th percentile) *n* = 20	C The best LVGLS (above 80th percentile) *n* = 9	*p* value
BASELINE				
Age (years)	64.8 ± 10.7	65.2 ± 11	73.2 ± 4.5	0.047^#^
Baseline LVGLS	− 5.2 ± 0.6	− 7.1 ± 0.7	− 10.6 ± 1.8	< 0.001^#^
Male sex, *n* (%)	17 (85)	17 (85)	7 (78)	0.87*
NYHA functional class	2.9 ± 0.5	2.6 ± 0.5	2.9 ± 0.4	0.24^#^
Ischemic HF etiology, *n* (%)	11 (55)	11 (55)	6 (67)	0.82*
QRS (ms)	178 ± 17.2	169.7 ± 20.2	168.1 ± 23	0.33^#^
LBBB, *n* (%)	17 (85)	13 (65)	5 (56)	0.19*
LVEDV (ml)	354.2 ± 157.6	252.7 ± 73.7	188.8 ± 56.8	< 0.001#
LVESV (ml)	276.7 ± 133.3	198.1 ± 64.5	133.2 ± 38.8	< 0.001#
LVEF (%)	22.1 ± 5.9	24 ± 4.5	33.3 ± 3.6	< 0.001#
AFTER CRT				
LVEF (%)	27.3 ± 6.3	35.1 ± 5	35 ± 6.5	< 0.001^#^
∆LVESV (%)	17.7 ± 20	33.3 ± 16.9	12.8 ± 16.3	0.006^#^
∆LVEF (absolute value)	5.2 ± 8.5	11.2 ± 6.2	1.7 ± 7.3	0.003^#^
∆LVEF (percentage value)	32.7 ± 49.1	51.3 ± 33.8	6 ± 22.4	0.033^#^
∆NYHA	0.95 ± 0.74	0.75 ± 0.94	0.83 ± 0.71	0.79^#^
∆ LVGLS (absolute value)	− 1.7 ± 2	− 1.1 ± 2.7	1.1 ± 3.6	0.08#
∆ LVGLS (percentage value)	136.9 ± 51.5	116.6 ± 39.2	91.7 ± 36.1	0.047#

*χ^2^ test for A vs. B vs. C

^#^Kruskal–Wallis test for A vs. B vs. C

**Table 4:** Comparison of “mid-range LVGLS” and “peripheral LVGLS”: before CRT implantation and after follow-up period

	“mid-range LVGLS” *n* = 20	“peripheral LVGLS” *n* = 29	*p* value
Baseline			
Age (years)	65 ± 11	67 ± 10	0.37
Male sex, *n* (%)	17 (85)	24 (83)	0.83
NYHA functional class	2.6 ± 0.5	2.9 ± 0.5	0.12
Ischemic HF etiology, *n* (%)	11 (55)	17 (58.6)	0.8
QRS (ms)	169.7 ± 20.2	175.2 ± 19.1	0.34
LBBB, *n* (%)	13 (65)	22 (75.9)	0.41
LVEDV (ml)	252 ± 74	302 ± 154	0.33
LVESV (ml)	198 ± 65	232 ± 131	0.47
LVEF (%)	24 ± 4.5	25.6 ± 7.4	0.4
After CRT			
LVEDV (ml)	198 ± 66	265 ± 141	0.07
LVESV(ml)	129 ± 46	192 ± 115	0.03
LVEF (%)	35.1 ± 5	29.7 ± 7.2	0.003
∆LVESV (%)	33.3 ± 16.9	16.2 ± 18.8	< 0.001
∆LVEF (absolute value)	11.2 ± 6.2	4.1 ± 8.2	< 0.001
∆LVEF (percentage value)	151.3 ± 33.8	124.4 ± 44	0.005
∆NYHA	0.75 ± 0.9	0.9 ± 0.7	0.61
∆ LVGLS (absolute value)	− 1.1 ± 2.7	− 0.8 ± 2.9	0.99
∆ LVGLS (percentage value)	116.6 ± 39.2	121.3 ± 44.8	0.92

**Fig. 4:**
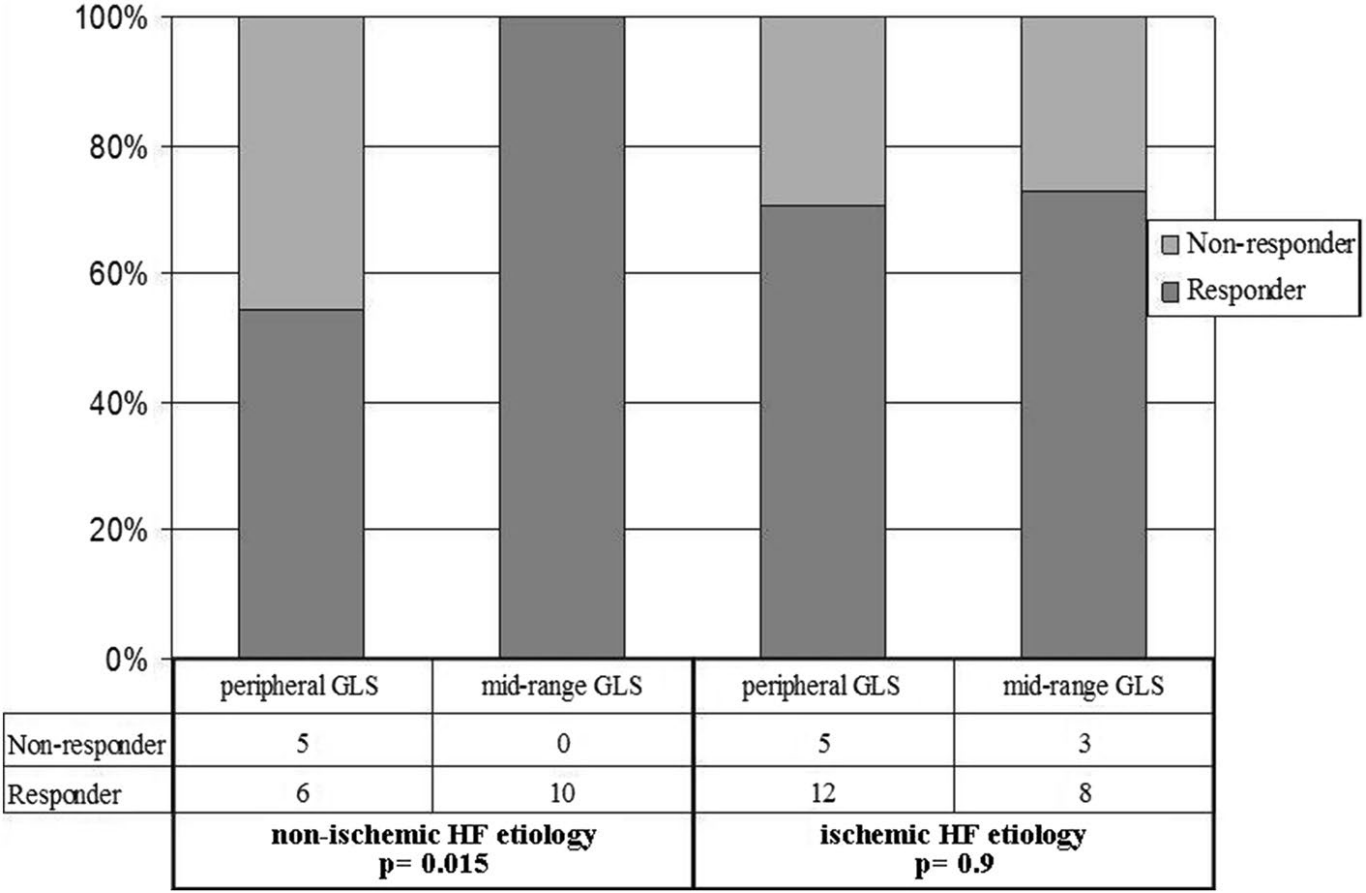
Response to CRT in non-ischemic/ischemic HF etiology with regard to LVGLS group (“peripheral LVGLS” and “mid-range LVGLS”)

Prediction of CRT response based on LVGLS values was particularly powerful in patients with non-ischemic HF etiology. In patients with non-ischemic etiology of HF, all “mid-range LVGLS” patients responded to CRT while among “peripheral LVGLS” there were 54.5% (6/11 patients) responders (*p* = 0.015; with Yates’s correction *p* = 0.0537). In patients with ischemic HF etiology, there was no difference in incidence of CRT responders between “peripheral LVGLS” and “mid-range LVGLS” (*p* = 0.9) (Fig. 4).

### Long-term follow-up

**Fig. 5:**
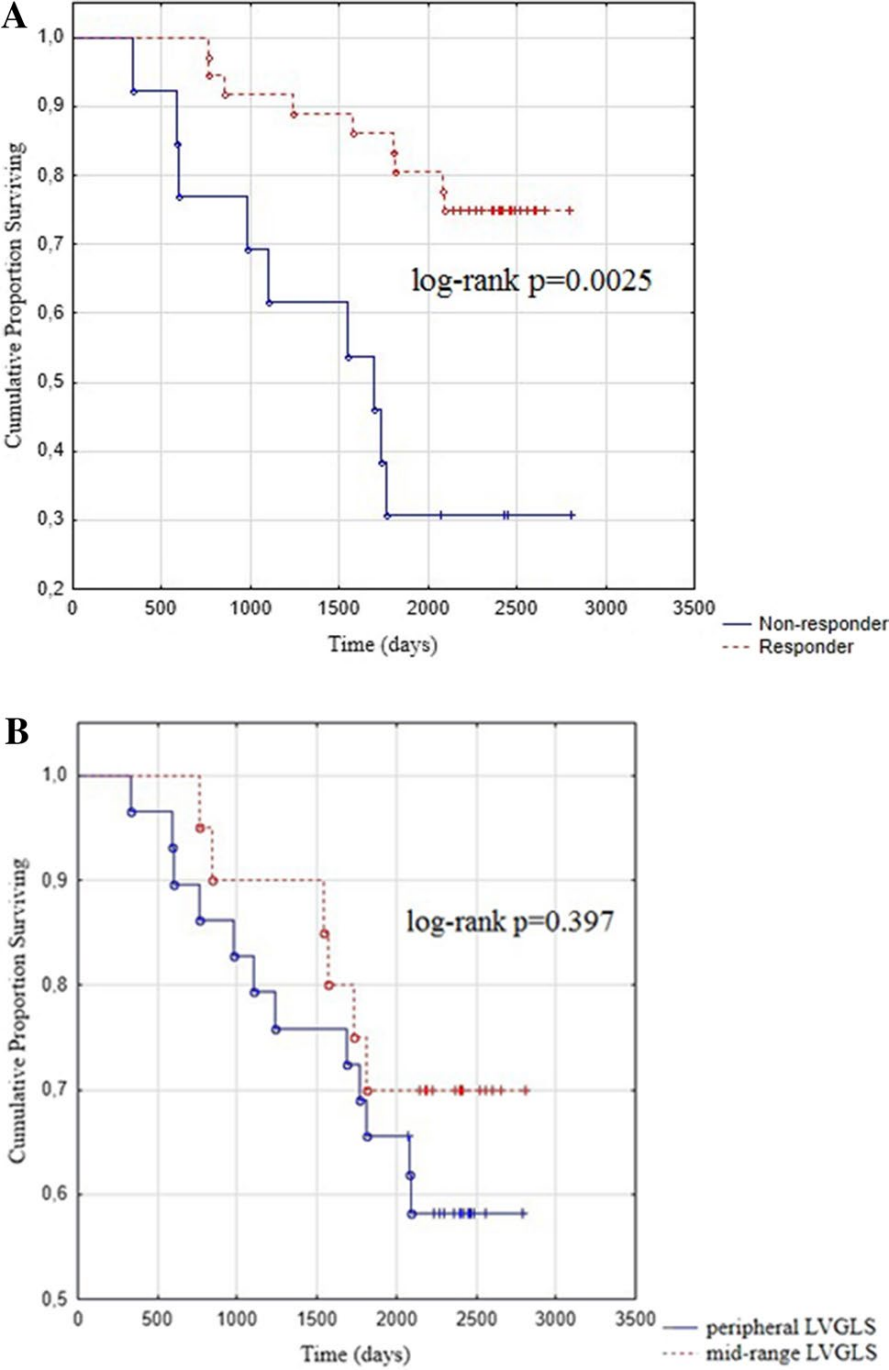
**a** Kaplan–Meier curves for all-cause mortality in the long-term follow-up according to category responder vs. non-responder. **b** Kaplan–Meier curves for all-cause mortality in the long-term follow-up according to category “peripheral LVGLS” and “mid-range LVGLS

The mean long-term follow-up was 2009 days (335–2808 days). During the long-term follow-up, 32 (65.3%) patients survived (one patient underwent heart transplantation) and 17 (34.7%) patients died. Comparison of the clinical and echocardiography data revealed significantly higher reduction of LVESV in survivals as compared to non-survivals obtained after the one-year follow-up (28.3 ± 17.5 vs. 14.4 ± 20.9 ml; *p* < 0.025). We did not observe any significant differences in other parameters. The Kaplan–Meier curves for all-cause mortality were calculated for the following categories: (a) responders and non-responders (log-rank *p* = 0.0025) (Fig. 5a); (b) “peripheral LVGLS” and “mid-range LVGLS” (log-rank *p* = 0.397) (Fig. 5b).

## Discussion

In this study, we intended to provide novel data concerning LV systolic function expressed as LVGLS, in the context of CRT. The main finding of the study is that baseline LVGLS may have a potential prognostic value in prediction CRT response with relationship of inverted J–shaped pattern.

LVGLS by speckle-tracking echocardiography characterizes global and regional LV function, LV scar burden, and myocardial viability. Previous studies showed LVGLS was associated with LV reverse remodeling [16] as well as with long-term outcome after CRT implantation [17]. Hasselberg et al. [18] suggested that CRT response by reverse remodeling may be dependent on improvement of longitudinal function. Gorcsan et al. [19] positively verified LVGLS as additive prognostic value in predicting CRT response. Our novel finding is that “mid-range LVGLS” values enable to select CRT responders, especially in non-ischemic HF etiology patients.

### Bermuda triangle: LVEF, LVGLS, and LVESV

We have found strong linear correlation between the baseline values LVGLS and LVEF (*r* = 0.64; *p* < 0.001), similar to other researchers [20]. There was no linear correlation between baseline LVGLS/LVEF and ∆LVESV after CRT (*r* = 0.09; *p* = 0.56 and *r* = − 0.25; *p* = 0.08, respectively). Echocardiographic response (∆LVESV) was associated with the improvement of LV performance expressed as ∆LVEF (*r* = 0.6; *p* < 0.001) and ∆LVGLS (*r* = 0.32; *p* = 0.028). Our results stay in line with the work of Menet et al. [21] who confirmed overmentioned correlations and showed ΔLVESV as a strong predictor of outcome following CRT.

### LVGLS and CRT

Important prognostic value of echocardiographic myocardial deformation parameters was confirmed in heart failure patients and CRT recipients [11]. Yan Ma and Delgado-Montero showed the ability of LVGLS as alone parameter in predicting positive effects of CRT. Respectively, LVGLS predicted echocardiographic response to CRT [16] and LVGLS was significantly associated with long-term outcome after CRT [17]. In the study [22] with large cohort of CRT patients, baseline LVGLS was independently associated with the combined endpoint. Kydd et al. [23] developed multiparametric predictive score (so-called “*p*-score”), incorporating LVGLS, offering a potential to predict CRT responders. Similar multiparametric echocardiographic score proposed by Park et al., including LVGLS, was helpful in selecting patients likely to undergo reverse remodeling and prediction clinical outcome [24].

Among the achievable studies dealing with longitudinal strain and CRT, final end-point was defined differently (LV reverse remodeling, long-term outcome or occurrence of ventricular arrhythmia) but relation”the better LVGLS, the better prognosis” remains in force. In our study, we have not found straight linear correlation between baseline LVGLS and ΔLVESV. In “mid-range LVGLS” group mean ΔLVESV was greater than in the patients with peripheral LVGLS values (33.3 ± 16.9% vs. 16.2 ± 18.8%; *p* = 0.00056). This group of 20 subjects was called “mid-range LVGLS” because among all the patients qualified to CRT implantation they had intermediate values of LVGLS. Remaining 29 patients represented extreme LVGLS values (either very good or very poor), therefore, we called this group “peripheral LVGLS”. Groups did not differ significantly in terms of demographics, clinical or echocardiographic characteristics.

The present study is the first to the best of our knowledge to evaluate the prognostic value of baseline LVGLS in non-linear manner. In the previous reports, better strain values were related with CRT benefits. Our results did not confirm the predictive value of LVGLS in this way. Patients with the highest LVGLS values in our study population (above 80th percentile; absolute LVGLS: < − 8.67%) reached average LVESV reduction. The shape of correlation line between baseline LVGLS and ∆LVESV resembles inverted “*J*-curve”. Our findings confirm the previous observations of QRS duration in patients with LBBB, a “U shaped” distribution resulted with non-responders clustered between 120 and 130 ms and above 180 ms [25]. Interestingly, for LVEF we were not able to set up comparable layout.

We disclosed non-linear prognostic value of LVGLS. Patients with mid-range baseline LVGLS were likely to benefit more than “peripheral LVGLS” patients. We have not found theoretic explanation of this fact in the literature. It does not seem to be a *bias* because other parameters and results keep in line with the previous studies, our patients reached comparable improvement in LVEF and LVESV reduction with similar percentage of responders [26], 27].

We have sought theoretical premise for explanation why patients with intermediate LVGLS values benefit the most. In patients with severely impaired systolic function, gain of synchronization will not contribute to overall heart’s performance. It seems to be “too late” and extremely decreased contractility/viability would not improve even after restoration of electrical dyssynchrony. In patients with better LV function, CRT profits would not be observed due to other reasons. In this group of patients, probability of extreme gain of LV function after restoration of LV synchrony seems lower due to underlying impairment of myocardium. In patients with ischemic cardiomyopathy, presence of scar, inactive part of LV, make unable to complete convalescence [28]. In patients with non-ischemic cardiomyopathy, the probability of CRT profits remains higher but we cannot rule out genetic conditioning which restrains absolute LV systolic function recovery.

The fiber direction of the subendocardial myocardial layer is mainly longitudinally oriented, while the fiber direction in the midmyocardial layer is mainly circular, although all are helically ordered [29]. Loss of longitudinal function from the subendocardial fibers is typical for ischemic damage and may explain why longitudinal function by LVGLS is less improved after CRT in patients with ischemic cardiomyopathy. Longitudinal functional reserve is present in non-ischemic patients, providing better chances for CRT response [18]. These findings support our results with regard to HF etiology.

We can find parallel with clinical condition expressed as NYHA functional class. In both fields, subjects with marginal quantities are less likely to respond favorably to CRT. ESC 2016 guidelines include NYHA class IV to CRT implantation but certain patients may be too sick to realize long-term mortality benefits from CRT [30]. Recent findings of Cimino et al. [31] suggest that end-stage HF patients, presenting before CRT with LVEF < 22.15%, may not benefit from the procedure after 6 months. On the other hand in NYHA class I, mortality benefit and symptom improvement [32] from CRT have not been demonstrated. Moreover, CRT might be harmful, in the European REVERSE substudy, the NYHA class I patients showed a trend toward worsened HF clinical composite response [33]. One can say, symptom severity correlates poorly with many measures of LV function; however, there are some papers supporting our assumption [34].

Similarly, QRS complex reflects the continuity of disease progression. Too healthy subjects, with narrow QRS do not respond to CRT. IVCD is associated with greater scar burden than LBBB in ischemic cardiomyopathy [35]. Patients with non-LBBB pattern show significantly less benefit from CRT than those with LBBB [36]. It seems that the perfect CRT candidate seems to be neither too sick nor healthy.

Our study also presents data of the long-term follow-up. We revealed that CRT response defined as ∆LVESV > 15% was positive survival prognostic factor. This is in accordance with the other studies [37, 38]. On the other hand, being peripheral/mid-range LVGLS influenced ∆LVESV. Therefore, we may suspect that the peripheral/mid-range LVGLS may also contribute in long term survival. Further studies with increased number of patients are necessary to confirm this hypothesis.

## Limitations

Small and heterogeneous sample is the most important limitation of the study. Study was not randomized. The quality of echocardiographic images is crucial for strain analysis; therefore, sometimes the image quality may be suboptimal. Post-implantation CRT optimization was not taken into consideration. Dyssynchrony analysis was performed but the results were not decisive and no single parameter was able to predict CRT response. Therefore, these results were not shown and we concentrated on the main aim of the study.

## Conclusions

Cardiac resynchronization therapy has a positive effect on overall outcome in HFrEF patients expressed as LV reverse remodeling. Baseline LVGLS may be an important marker in predicting response to CRT with the relationship of inverted J-shaped curve pattern. Patients with intermediate LVGLS values are more likely to respond positively to CRT, especially in non-ischemic etiology of HF.
